# Supplementary material for: SAP-X2C: Optimally-Simple Two-Component Relativistic Hamiltonian with Size-Intensive Picture Change
Source: J Chem Theory Comput. 2026 Mar 26;22(7):3443–52. doi: 10.1021/acs.jctc.6c00032 (PMC13085237; doi:10.1021/acs.jctc.6c00032)
Supplement: Supplementary file 1 [file ct6c00032_si_001.zip › supporting-information/supporting-information.pdf]

SAP-X2C: Optimally-Simple Two-Component Relativistic Hamiltonian With Size-Intensive Picture Change

Kshitijkumar A. Surjuse and Edward F. Valeev*

Department of Chemistry, Virginia Tech, Blacksburg, VA 24061

E-mail: efv@vt.edu

March 9, 2026

S1 Molecular geometries

The Supporting Information accompanying this publication includes a .zip file that contains:

1. Files containing all molecular geometries used for the energy calculations in this work, in `.xyz` format.
2. `.xyz` files for Xe lattice fragments. The recipe to construct these fragments is described below in Section S2.

S2 Construction of Xe lattice fragments

Xe forms a face-centered cubic (FCC) lattice structure with a unit cell of length $a = 6.2023$ Å.[1] The construction of crystal fragments follows a simple recipe, and can primarily be formed by an alternating arrangement of yz plane tiling from Figures S1a and S1b along

x-axis. Let us first look at Figure S1a (L1) that tiles yz planes at a given x. The distance between any two Xe atoms along the y or z axis is the unit cell size a, and the adjacent (parallel) 1D arrangements (or 1D layers) are separated by $a/2$, depicted by the dotted lines in Figure S1. In Figure S1a, it can be seen that the smallest square formed by the dotted lines around the origin constitutes the face of the FCC unit cell. Figure S1b shows the second type of arrangement (L2) that tiles yz planes needed for the FCC lattice construction. Now, if we arrange L1 at $x = 0$, L2 at $x = a/2$ and L1 again at $x = a$, it can be seen that the cube $(0, 0, 0) \rightarrow (a, a, a)$ forms the FCC unit cell. In this work, to keep the arrangement of atoms symmetric around the atom at the origin, we construct the fragments by arranging L1s at $x = Na$ and L2s at $x = (N + 1)a/2$, where $n \in .... - 2, -1, 0, 1, 2, ...$, and then taking cubic slices between $(-Na/2, -Na/2, -Na/2) \rightarrow (Na/2, Na/2, Na/2)$.

References

(1) Sears, D. R.; Klug, H. P. Density and Expansivity of Solid Xenon. *J. Chem. Phys.* **1962**, *37*, 3002–3006.

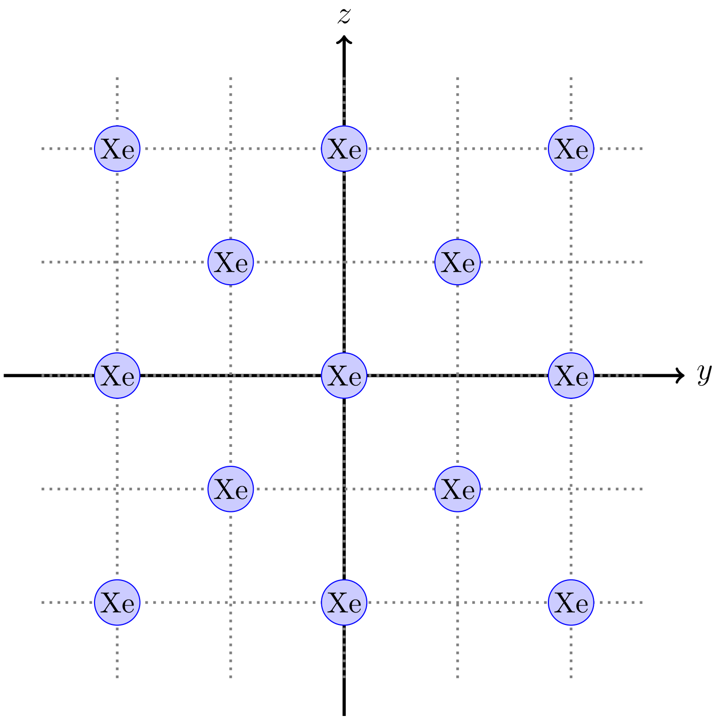

(a) Layer type-1 of Xe atoms in the yz-plane (L1).

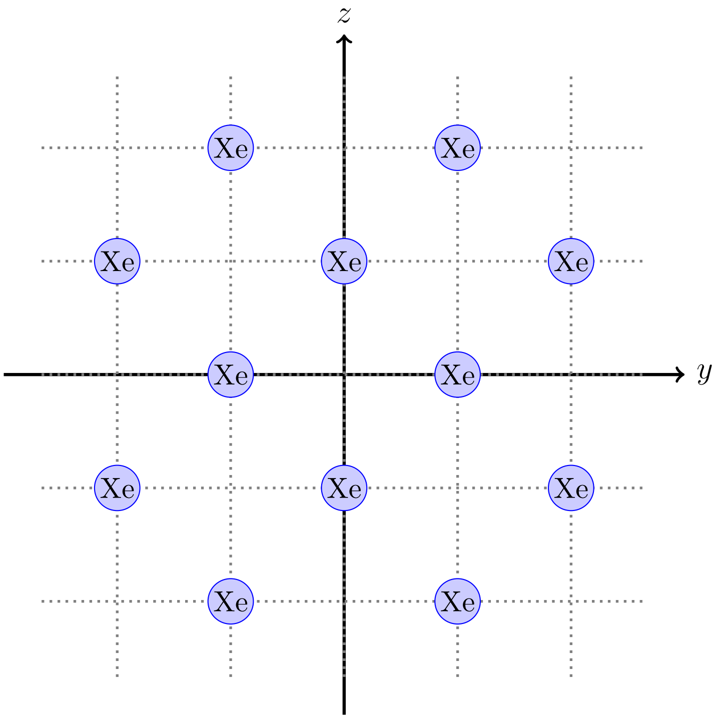

(b) Layer type-2 of Xe atoms in the yz-plane (L2).

Figure S1
